# Stingless bee honey protects against lipopolysaccharide induced-chronic subclinical systemic inflammation and oxidative stress by modulating Nrf2, NF-κB and p38 MAPK

**DOI:** 10.1186/s12986-019-0341-z

**Published:** 2019-02-27

**Authors:** Yazan Ranneh, Abdah Md Akim, Hasiah Ab Hamid, Huzwah Khazaai, Abdulmannan Fadel, Ayman M. Mahmoud

**Affiliations:** 1 Department of Nutrition and Dietetics, Faculty of Medicine and Health Sciences, Universiti Putra Malaysia, UPM, 43400 Serdang, Selangor Malaysia; 2 Department of Biomedical Sciences, Faculty of Medicine and Health Sciences, Universiti Putra Malaysia, UPM, 43400 Serdang, Selangor Malaysia; 3 School of Food Science and Nutrition, University of Leeds, Leeds, UK; 4 Physiology Division, Zoology Department, Faculty of Science, Beni-Suef University, Beni-Suef, Egypt

**Keywords:** Chronic subclinical systemic inflammation, Stingless bee honey, Lipopolysaccharide, Oxidative stress, Cytokines

## Abstract

**Background:**

Epidemiological and experimental studies have extensively indicated that chronic subclinical systemic inflammation (CSSI) and oxidative stress are risk factors for several chronic diseases, including cancer, arthritis, type 2 diabetes, and cardiovascular and neurodegenerative diseases. This study examined the protective effect of stingless bee honey (SBH) supplementation against lipopolysaccharide (LPS)-induced CSSI, pointing to the possible involvement of NF-κB, p38 MAPK and Nrf2 signaling.

**Methods:**

CSSI was induced in male Sprague Dawley rats by intraperitoneal injection of LPS three times per week for 28 days, and SBH (4.6 and 9.3 g/kg/day) was supplemented for 30 days.

**Results:**

LPS-induced rats showed significant leukocytosis, and elevated serum levels of CRP, TNF-α, IL-1β, IL-6, IL-8, MCP-1, malondialdehyde (MDA) and 8-hydroxy-2′-deoxyguanosine (8-OHdG), accompanied with diminished antioxidants. Treatment with SBH significantly ameliorated inflammatory markers, MDA and 8-OHdG, and enhanced antioxidants in LPS-induced rats. In addition, SBH decreased NF-κB p65 and p38 MAPK, and increased Nrf2 expression in the liver, kidney, heart and lung of LPS-induced rats. Furthermore, SBH prevented LPS-induced histological and functional alterations in the liver, kidney, heart and lung of rats.

**Conclusion:**

SBH has a substantial protective role against LPS-induced CSSI in rats mediated via amelioration of inflammation, oxidative stress and NF-κB, p38 MAPK and Nrf2 signaling.

## Background

Inflammation is defined as a biological protective response to counteract stressor and harmful pathogenic stimuli [1]. The inflammatory process, which could be clinical or subclinical, consists of a tremendously complex interplay of immune cells, blood vessels and molecular mediators [2]. The clinical features of inflammation have been traditionally identified as cardinal signs, including redness, swelling, pain and loss of function [3]. However, dysregulation of inflammatory suppression occurs in autoinflammatory diseases due to pointless events of inflammation [4]. The presence of inflammation is inferred if nuclear factor-kappaB (NF-κB) is activated and pro-inflammatory cytokines production is increased [5]. Hence, various pathological conditions are associated with increased levels of the inflammatory mediators despite the absence of clinical signs. These conditions have been characterized in different terms namely, chronic subclinical systemic inflammation (CSSI), low-grade chronic inflammation, mini-inflammation and meta-inflammation (metabolically-triggered inflammation) [6]. The exposure of cells to oxidative stress and metabolic malfunction triggers CSSI which plays an important role to restore homeostasis [7]. Oxidative stress and inflammation have been found to have a positive feedback mechanism that arises at systemic or local level [1]. Hence, the crosstalk between CSSI and oxidative stress has been thought to be involved in the pathogenesis of type 2 diabetes, cancer, metabolic syndrome, obesity, arthritis, osteopenia, and neurodegenerative and cardiovascular diseases [8–14].

The initial step of activated CSSI is the release of pro-inflammatory cytokines and mediators. The elevated levels of C-reactive protein (CRP), interleukin (IL)-6 and tumor necrosis factor (TNF)-α have been used clinically to determine the presence of CSSI [15]. TNF-α and IL-1β have shown a perpetuation of signaling cascade, while monocyte chemoattractant protein (MCP)-1 functions as a chemoattractant molecule for leukocytes, recruiting neutrophils and monocytes to the site of damage and inflammation [16]. At the same time, activation of NF-κB is concomitant with increased production of reactive oxygen species (ROS) which ultimately lead to oxidative damage of the cellular components, protein, lipids and DNA. Despite the presence of efficient anti-inflammatory drugs, such as, corticosteroids and non-steroidal anti-inflammatory drugs (NSAIDs), their long-term administration has been associated with numerous side effects [17]. Therefore, new anti-inflammatory agents with fewer or no side effects are needed.

Healthy functional foods rich in bioactive compounds have been linked with lower CSSI [18], and a strong evidence of epidemiological studies has indicated that the consumption of polyphenol-rich foods may modulate CSSI [19]. Since floral nectar is rich in polyphenols, honey may be considered as a functional transporter of medicinal plants’ polyphenols [20]. In this context, stingless bee honey (SBH) has been found to be rich in polyphenols when compared with other types of honey [21–23]. Recently, emerging experiments have indicated that SBH possesses several beneficial effects under pathological conditions, rendering it a promising functional food with anti-oxidant, anti-bacterial, anti-cancer and anti-inflammatory properties [24–28]. Therefore, SBH could be a reasonable dietary intervention to prevent or treat CSSI-related diseases. The current study aimed to investigate the ameliorative potential of SBH in lipopolysaccharide (LPS)-induced CSSI with an emphasis on oxidative stress, and NF-κB, p38 mitogen-activated protein kinase (MAPK) and nuclear factor erythroid 2–related factor 2 (Nrf2) signaling in rats.

## Methods

### Reagents and kits

LPS (derived from *Escherichia coli* 055:B5) and all solvents and reagents were supplied by Sigma-Aldrich (St. Louis, MO, USA). CRP, reduced glutathione (GSH), glutathione-S-transferase (GST), glutathione peroxidase (GPx), malondialdehyde (MDA) and 8-hydroxy-2′-deoxyguanosine (8-OHdG) assay kits were purchased from Melsin Medical Co. (Changchun, Jilin, China). IL-6, IL-8, TNF-α, IL-1β, MCP-1, NF-κB p65, p38 MAPK and Nrf2 sandwich ELISA kits were supplied by Wuhan Fine Biotech (Wuhan, China).

### SHB sample and LC-MS/MS analysis

SBH, produced by *Trigona*, was supplied by local honey accumulator in Johor Bahru state, Malaysia. The botanical origin of SBH was based on different types of flora, including Forest Mangorve (*Acacia mangium*), Rambutan (*Nephelium lappaceum*), Longan (*Dimocarpus longan*) and Belimbing (*Averrhoa carambola*). SBH was kept in sterile air-tight-glass bottles at 15 °C to prevent any moisture absorption during the collection and experimental time.

Each peak of SBH separated using LC-ESI-MS/MS was carried out using AB Sciex 5500Q Trap LC/MS-MS system consisting of degasser, binary pump, auto sampler and column heater. The column vent was coupled to an Agilent 1290 series UHPLC mass spectrometer (Agilent Technologies, Palo Alto, CA, USA), equipped with an ESI ion source. Data acquisition and mass spectrometric evaluation were performed in the laboratory computer of our institute using Sciex Analyst software (version 1.5). For chromatographic separation, a reversed phase C18 column (Phenomenex Synergi Fusion; 100 mm X 2.1 mm, particle size 3 μm) was used with working temperature 35 °C (Waters, MA, USA). The injection volume was 10 μL. The mobile phase solvents contained solvent A (water with 0.1% formic acid and 5 mM ammonium formate) and solvent B (acetonitrile with 0.1% formic acid and 5 mM ammonium formate). The following gradient run program was applied: 5–95% B: 0.01–10.0 min, holding for 2 min and back to 10% B in 0.1 min and re-equilibration for 3 min. The overall flow rate was 250 μl/min. The following operating conditions were used during all MS experiments: for (ESI) Turbo interface operating was in a negative mode that has been proven to be more selective and efficient in characterizing phenolic compounds [29], the capillary voltage was set to 4.5 kV, the drying temperature to 500 °C, the nebulizer pressure to 40 psi and the drying gas flow to 100/min. The single ion monitoring (SIM) modality was performed to quantify the molecular ions of phenolic compounds. The SIM analysis, in this experiment, was scanned in the range of 100–1000 m/z for full scan and 50–1000 m/z for MS/MS scan. The mass fragmentation was done based on ACD/Labs advanced chemometrics mass fragmentation predictive software.

The phenolic acids and flavonoids were identified using a combination of liquid chromatography with mass spectrometry (ESI-LC-MS/MS) based on their ultraviolet (UV) spectra, retention time, mass spectra and by comparing with our standard library information that includes 500 established phenolic compounds.

### Calculation of SHB dose for the in vivo experiments

Consuming honey for two weeks continuously at a dose of 1.2 g/kg in healthy subjects has shown an improvement in the antioxidant status [30]. In addition, a clinical trial has proven that the consumption of a diet supplemented with 1.5 g/kg increased the level of antioxidants [31]. Nonetheless, there are various in vivo experiments that designed the dosage of honey treatment based on isometric scaling (direct extrapolation on g/kg basis). Tualang and Gelam honeys were administrated orally to rodents at doses range from 1 to 2 g/kg/day which was used previously in clinical trials [32, 33]. In the current investigation, we calculated the dose of SBH according to the Food and Drug Administration (FDA) guidelines. Based on previous clinical trial [31], the consumed honey at a dose of 1.5 g/kg was converted mathematically into the animal equivalent dose using allometric scaling. The animal equivalent doses of biological compounds were calculated using the following formula:

$$ AED\ \left(\frac{mg}{kg}\right)= HED\left(\frac{mg}{kg}\right)x\  Km\  ratio $$

AED: Animal Equivalent Dose, HED: Human Equivalent Dose and K_m_ ratio is a correction factor estimated by dividing the average body weight (kg) of species to its body surface area (m^2^). Thus, rats correction factor is equal to 6.2 [34]. These guidelines are the most acceptable for human to animal dose conversion for biological active agents [35]. Based on this equation, the AED is 9.3 g/kg.

### Experimental animals and treatments

Animal ethics approval was obtained from the Institutional Animal Care and Use Committee (IACUC) of University Putra Malaysia prior to starting the study (UPM/IACUC/AUP-R007/2017). A total of 36 pathogen-free 7-weeks old male Sprague-Dawley (SD) rats, weighing 250–290 g, were obtained from University Putra Malaysia and used in the present study. The rats were housed in cages under standard conditions with 12 h–12 h light-dark cycle, and normal temperature (22–24 °C) and humidity (40–60%). The rats were given free access to a standard rodent chow and water *ad libtium* and were acclimatized for two weeks before starting the experiment.

**Fig. 1:**
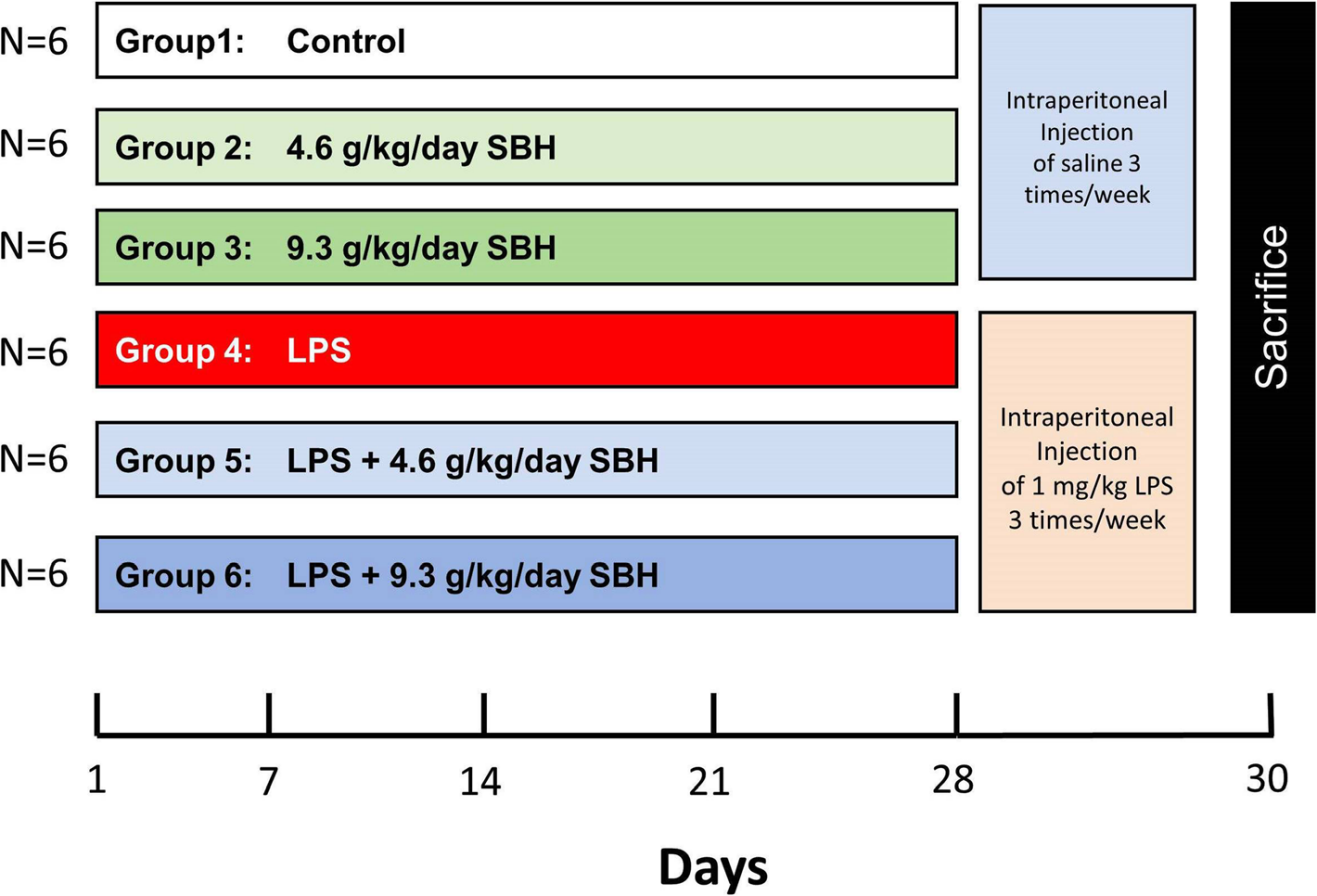
A schematic diagram showing the experimental design. SBH, Stingless bee honey; LPS, Lipopolysaccharide

The rats were divided randomly into six groups (*n* = 6) as following (Fig. 1):

Group 1 (Control): rats received distilled water via oral gavage for 30 days and intraperitoneal (ip) injection of physiological saline 3 times/week for 28 days.

Group 2 (4.6 g/kg SBH): rats received 4.6 g/kg SBH dissolved in distilled water via oral gavage for 30 days and ip injection of physiological saline 3 times/week for 28 days.

Group 3 (9.3 g/kg SBH): rats received 9.3 g/kg SBH dissolved in distilled water via oral gavage for 30 days and ip injection of physiological saline 3 times/week for 28 days.

Group 4 (LPS): rats received distilled water via oral gavage for 30 days and ip injection of LPS (1 mg/kg) dissolved in physiological saline 3 times/week for 28 days.

Group 5 (LPS + 4.6 g/kg SBH): rats received 4.6 g/kg SBH dissolved in distilled water via oral gavage for 30 days and ip injection of LPS (1 mg/kg) dissolved in physiological saline 3 times/week for 28 days.

Group 6 (LPS + 9.3 g/kg SBH): rats received 9.3 g/kg SBH dissolved in distilled water via oral gavage for 30 days and ip injection of LPS (1 mg/kg) dissolved in physiological saline 3 times/week for 28 days.

### Measurement of body weight, food intake and survival rate

Body weight and food intake were measured each five days for each animal using electric digital scale. Pre-weighed foods were given to the rats on the first day of experiment, then food intake was measured daily by subtracting the weight of food bin from the previous day. The survival percentage of the animals was carried on through a close observation and follow-up. Kaplan-Meier survival curve was used to analyze the estimated probability.

### Termination procedure and hematological measurements

After 30 days of the experiment, the animals were anesthetized using ip injection of sodium pentobarbital (70 mg/kg) and were sacrificed by withdrawing the blood via cardiac puncture. Half of the blood was collected on EDTA to measure the white blood cells (WBCs) count using an automated blood cell analyzer (Abbott Cell Dyn 1800, Abbott, Illinois, USA), while the other half was used to separate serum. The rats were dissected immediately, and liver, kidney, heart and lung were surgically excised and washed with ice-cold phosphate buffered saline (PBS). Samples from each tissue were collected on 10% neutral buffered formalin while others were minced and homogenized (10% *w*/*v*) in ice-cold 50 mM sodium dihydrogen phosphate buffer (pH = 7.5) containing 1 mM EDTA and 1% Triton-X. The homogenates were centrifugated at 10,000 g for 20 min at 4 °C, and supernatants were collected and stored at − 80 °C. The protein content in the tissue homogenates was estimated according to the method of Lowry et al. [36]. The homogenates were kept frozen at − 80 °C for further analysis.

### Determination of inflammatory mediators

Serum levels of CRP, IL-1β, IL-6, IL-8, TNF-α and MCP-1 were determined using specific ELISA kits according to the manufacturer’s instructions.

### Determination of NF-κB p65, p38 MAPK, and Nrf2

NF-κB p65, p38 MAPK and Nrf2 were determined in the homogenate samples of liver, kidney, heart and lung using specific ELISA kits according to manufacturer’s instructions.

### Determination of antioxidants and oxidative stress markers

GSH, GST, GPx, 8-OHdG and MDA were determined in serum samples using commercially available specific assay kits.

### Determination of liver, kidney and heart function markers

Alkaline phosphatase (ALP), aspartate aminotransferase (ALT), alanine aminotransferase (AST), creatinine, urea and creatine kinase (CK) were determined in the serum samples using Hitachi 900 Auto Analyzer (Roche Diagnostics, Switzerland), according to the manufacturer’s protocol.

### Histological examination

The fixed samples from liver, kidney, heart and lung were embedded in paraffin blocks to process for routine histological evaluation using hematoxylin and eosin (H&E) staining [37]. A skilled pathologist who was blind to the study evaluated the pathological changes and scored inflammation using Microscope Imaging Cell Software connected with Olympus light microscope BX40 (Olympus Optical Co., Japan). Inflammation scoring was conducted based on the following criteria: score 0 = normal tissue (absence of inflammation), score 1 = the inflammatory cells and tissue damage are present in less than 25% of field view, score 2 = inflammation and tissue damage involved in 25 to 50% of field view, score 3 = inflammation and tissue damage involved in 50 to 75% of field view, and score 4 = inflammation and tissue damage involved in more than 75% of field view.

### Statistical analysis

Quantitative results were expressed as mean ± standard error of the mean (SEM). The analysis of data was performed using GraphPad Prism software (Prism 7.0, GraphPad Software Inc., CA, USA). The normality of data (Kolmogorove-Smirnov test) and homogeneity of variance were performed. The significance level was tested using one-way analysis of variance (ANOVA) followed by a post hoc Tukey’s test. A *P* value less than 0.05 was considered significant.

## Results

### Identification of polyphenols in SBH by LC- ESI-MS/MS

**Fig. 2:**
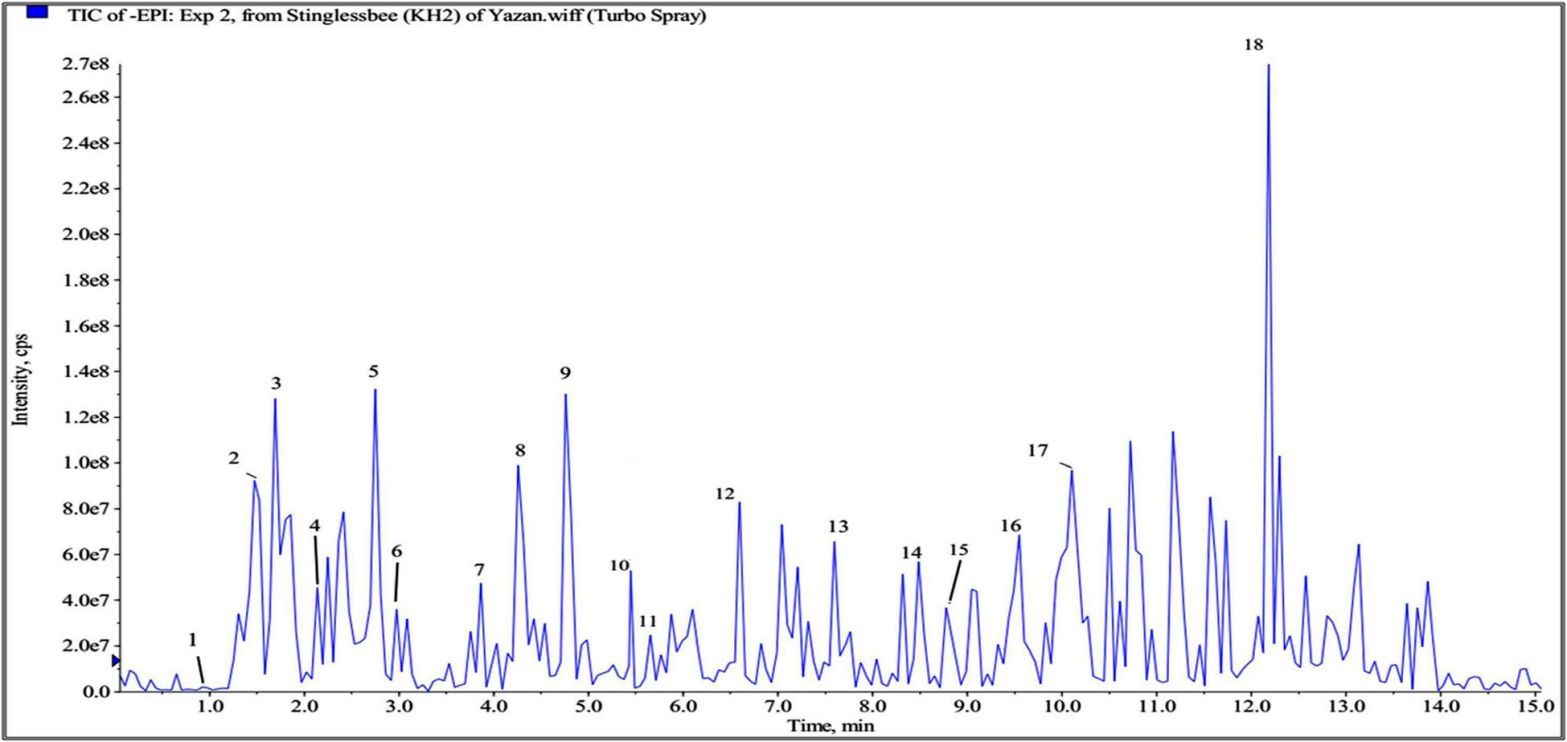
LC-MS chromatogram at 280 nm of SBH

Based on the optimization conditions of LC-ESI-MS/MS, SBH was subjected to identify the polyphenols. By searching in our standard library information (e.g. Peak retention times, UV spectrum, [M-H] (m^2^) and ESI-MS/MS data), a total of 18 phenolic compounds, consisting of around 8 phenolic acid compounds and 5 flavonoids, were investigated with range of 0.8–12.4 retention times (RT) in total ion chromatogram (Table 1 and Fig. 2). Peaks 1, 2, 3, 4, 10, 12, 14, and 18 referred to phenolic acid class while peaks 5, 7, 8, 13 and 16 belong to the flavonoids class. The other 5 unknown compounds were not identified under the same conditions. As shown in Fig. 2 and Table 1, gallic acid, caffeic acid, chrysin, cinnamic acid, 2-hydroxycinnamic acid, kaempferol, p-coumaric acid, catechin, quercetin-3-O-rutinosid, caffeic acid phenethyl ester and 4-hydroxybenzoic acid were identified in SBH. Notably, caffeic acid, caffeic acid phenethyl ester, cinnamic acid, 2-hydroxycinnamic acid, and p-coumaric acid are classified as hydroxycinnamic acids.

**Table 1:** Phenolic compounds identified in SBH by LC-MS/MS with ESI

Peak No	RT (min)	[*M*-H]^−^ (Frag. MS^2^ *m/z*)	Molecular formula	Compounds
1	0.92	169 (125)	C_7_H_6_O_5_	Gallic acid
2	1.3	179 (135)	C_9_H_8_O_4_	Caffeic acid
3	1.74	283.8 (241, 221, 179)	C_17_H_16_O_4_	Caffeic acid phenethyl ester
4	2.09	197 (182, 147)	C_9_H_10_O_5_	Syringic acid
5	2.75	289 (244.9, 175, 147)	C_15_H_14_O_6_	Catechin
6	2.97	775 (715)	NI	Unknown
7	3.87	269 (251, 225, 205)	C_15_H_10_O_5_	Apigenin
8	4.2	253 (209, 193, 178)	C_15_H_10_O_4_	Chrysin
9	4.77	221	NA	Unknown
10	5.3	147 (118, 129, 102)	C_9_H_8_O_2_	Cinnamic acid
11	5.67	162	NI	Unknown
12	6.5	165 (147, 119)	C_9_H_8_O_3_	2-Hydroxycinnamic acid
13	7.6	285 (175, 151, 133)	C_15_H_10_O_6_	Kaempferol
14	8.5	163 (119)	C_9_H_8_O_3_	*P* coumaric acid
15	8.9	959 (913)	NI	Unknown
16	9.6	609 (301)	C_9_H_8_O_3_	quercetin-3-O-rutinosid
17	10.2	614 (478, 452, 342)	NI	Unknown
18	12.4	137 (93)	C_7_H_6_O_3_	4-Hydroxybenzoic acid

NI, Not identified; RT, Retention time; [*M*-H]^−^, Molecular mass of honey extract on the loss of one proton measured by SIM

### Effect of SBH on body weight, food intake and survival rate in LPS-induced rats

**Fig. 3:**
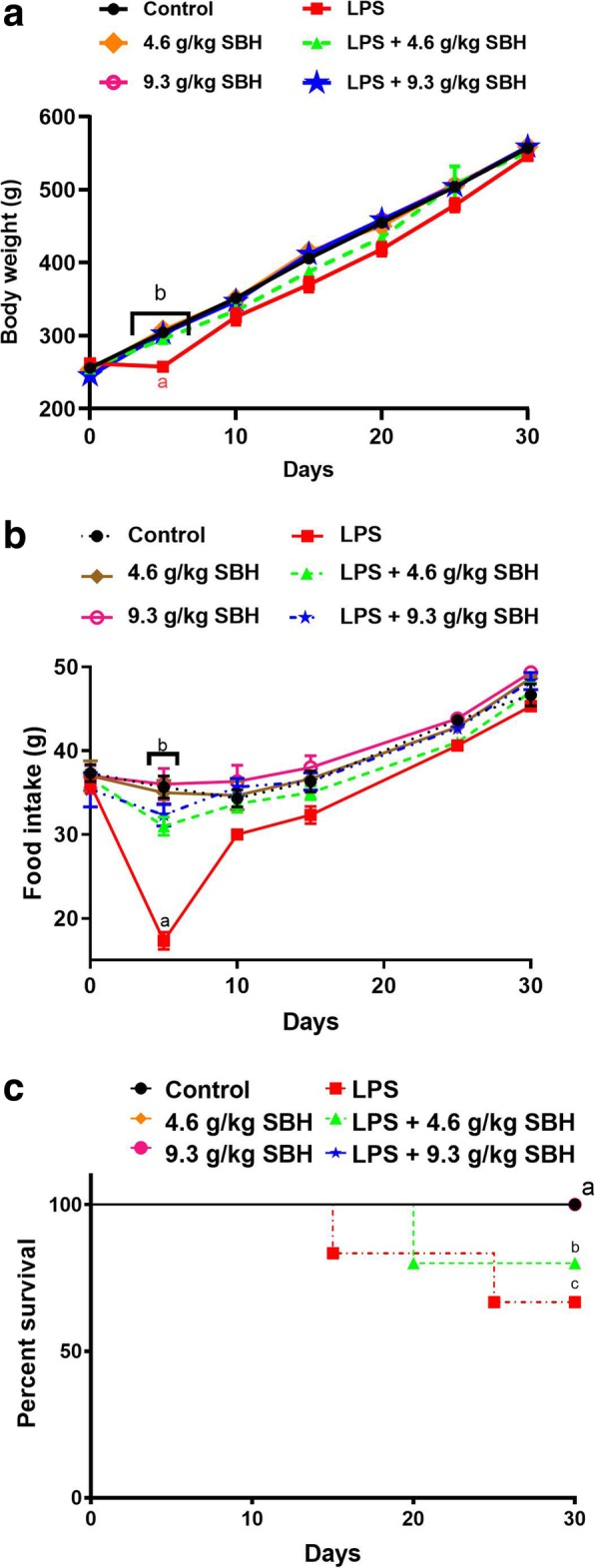
Effect of SBH on (**a**) body weight changes, (**b**) food intake and (**c**) survival rate in control and LPS-induced rats. Data are mean ± standard error of the mean (*n* = 6). Significantly different values are indicated by different superscripts

Oral supplementation of SBH didn’t induce significant changes in the body weight of normal rats as represented in Fig. 3A. LPS-induced rats showed a significant (*P* < 0.05) reduction in body weight during the first week, but after 10 days of the experiment this reduction wasn’t significant when compared with the control group. On the other hand, LPS-induced rats treated with SBH exhibited non-significant changes in body weight (Fig. 3A). Moreover, a notable decrease in food consumption with 18 g was observed in the LPS group, corresponding with the same day of weight reduction, but this reduction was recovered to reach 30 g on day 10, as shown in Fig. 3B. The rats in LPS + 4.6 g/kg SBH group exhibited less food intake when compared with the control group; however, this reduction was not significant (Fig. 3B).

The survival curves of all animal groups during 30 days of the experiment are demonstrated in Fig. 3C. The survival rate was 100% in the control and SBH-supplemented, and LPS-induced rats treated with 9.3 g/kg SHB groups. In contrast, the survival rate of LPS-induced rats supplemented with 4.6 g/kg SBH (83.4%) was significantly less than the control group. Two rats from LPS group were died at different intervals, showing a significant (*P* < 0.05) reduction in survival rate (66.7%). The difference in survival rate between untreated and 9.3 g/kg SBH treated LPS-induced was significant (*P* < 0.05).

### Effect of SBH on hematological changes in LPS-induced rats

**Fig. 4:**
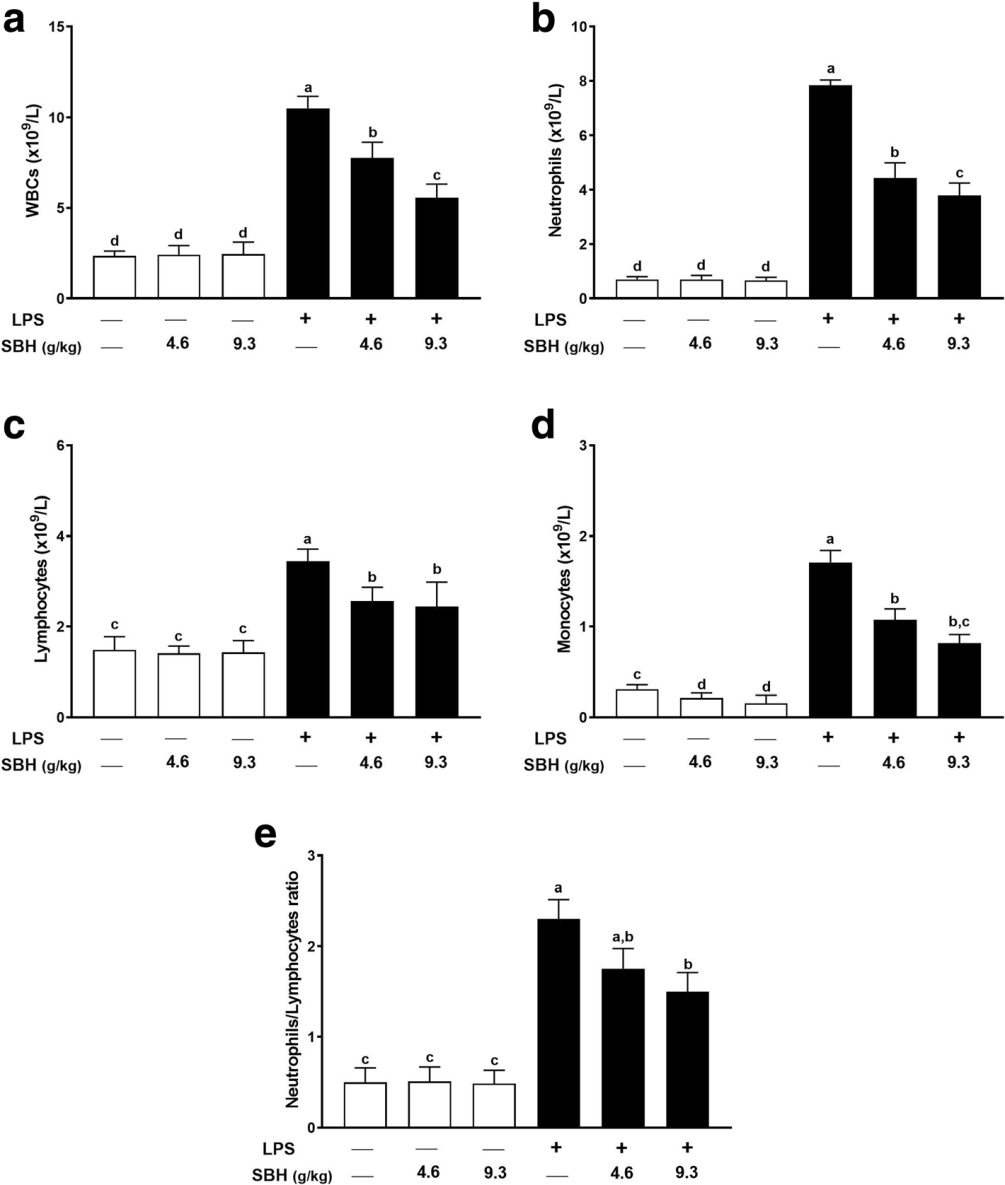
SBH ameliorates LPS-induced (**a**) leukocytosis, (**b**) neutrophilia, (**c**) lymphocytosis, (**d**) monocytosis and (**e**) neutrophils/lymphocytes ratio in rats. Data are mean ± standard error of the mean (*n* = 6). Significantly different values are indicated by different superscripts

The total number of WBCs in LPS-induced rats was significantly higher than that of the normal group as depicted in Fig. 4A. Similarly, neutrophils (Fig. 4B), lymphocytes (Fig. 4C), monocytes (Fig. 4D) and neutrophils/lymphocytes ratio (Fig. 4E) were significantly increased in LPS group when compared with the control group. Treatment with either dose of SBH significantly ameliorated both the total and differential WBCs count in LPS-induced rats (Fig. 4). Normal rats received either dose of SBH didn’t demonstrate any notable changes in WBCs when compared with the control group (Fig. 4).

### Effect of SBH on inflammatory cytokines and mediators in LPS-induced rats

**Fig. 5:**
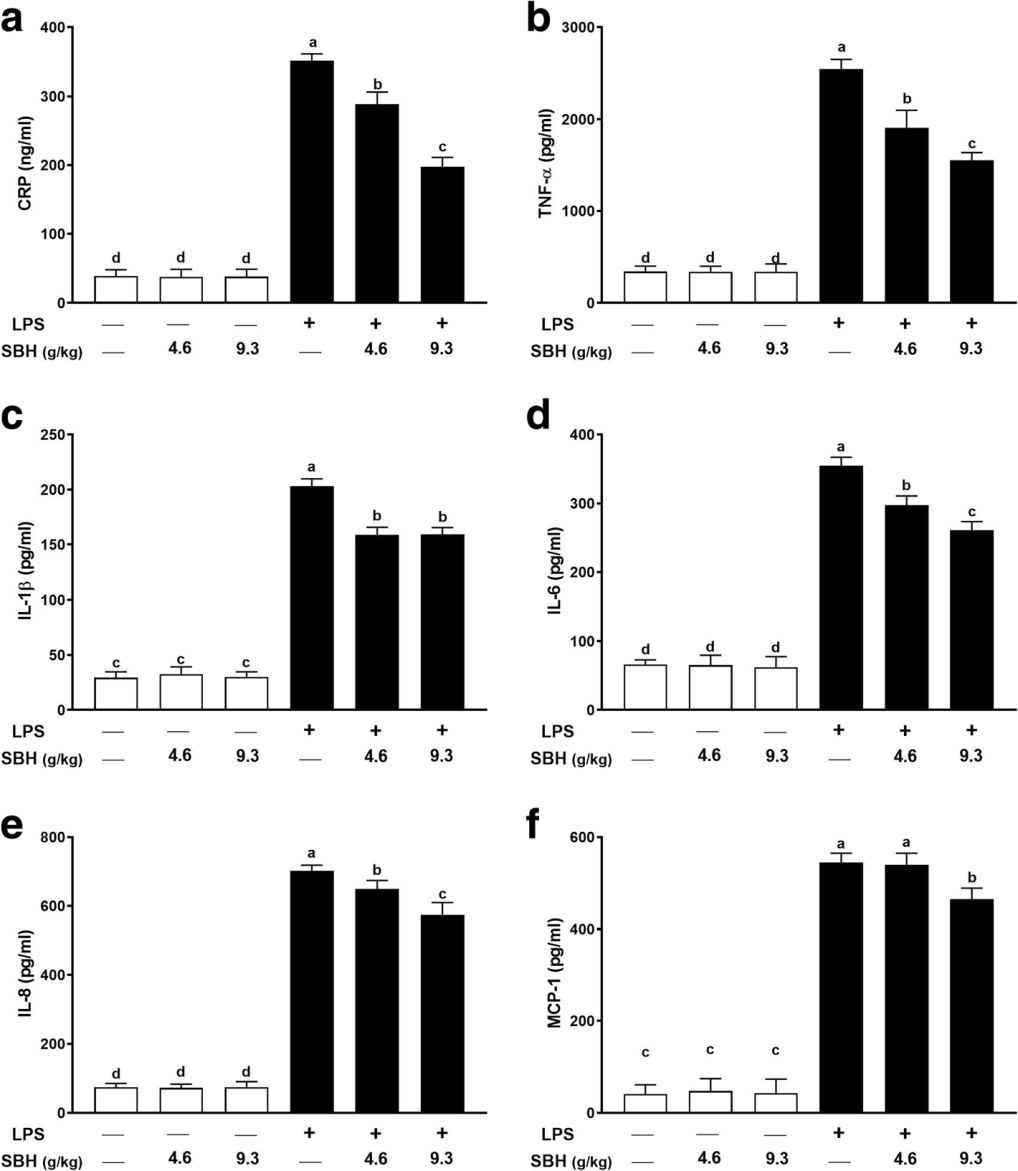
SBH attenuates LPS-induced inflammation in rats. Treatment with 4.6 and 9.3 g/kg SBH for 30 days significantly reduced serum levels of (**a**) CRP, (**b**) TNF-α, (**c**) IL-1β, (**d**) IL-6, (**e**) IL-8 and (**f**) MCP-1 in LPS-induced rats. Data are mean ± standard error of the mean (*n* = 6). Significantly different values are indicated by different superscripts

Serum levels of CRP (Fig. 5A), TNF-α (Fig. 5B), IL-1β (Fig. 5C), IL-6 (Fig. 5D), IL-8 (Fig. 5E) and MCP-1 (Fig. 5F) were significantly (*P* < 0.05) increased in LPS-induced rats when compared with the control group. LPS-induced rats treated with both doses of SBH exhibited significantly ameliorated serum levels of CRP, TNF-α, IL-1β, IL-6 and IL-8. However, the daily supplementation of 4.6 g/kg SBH didn’t reduce the levels of MCP-1, whereas the higher dose (9.3 g/kg) significantly (*P* < 0.05) decreased serum MCP-1 in LPS-induced rats (Fig. 5F). Oral administration of 4.6 and 9.3 g/kg SBH didn’t induce significant changes in serum inflammatory mediators in normal rats (Fig. 5).

### Effect of SBH on NF-κB p65 and p38 MAPK in LPS-induced rats

**Fig. 6:**
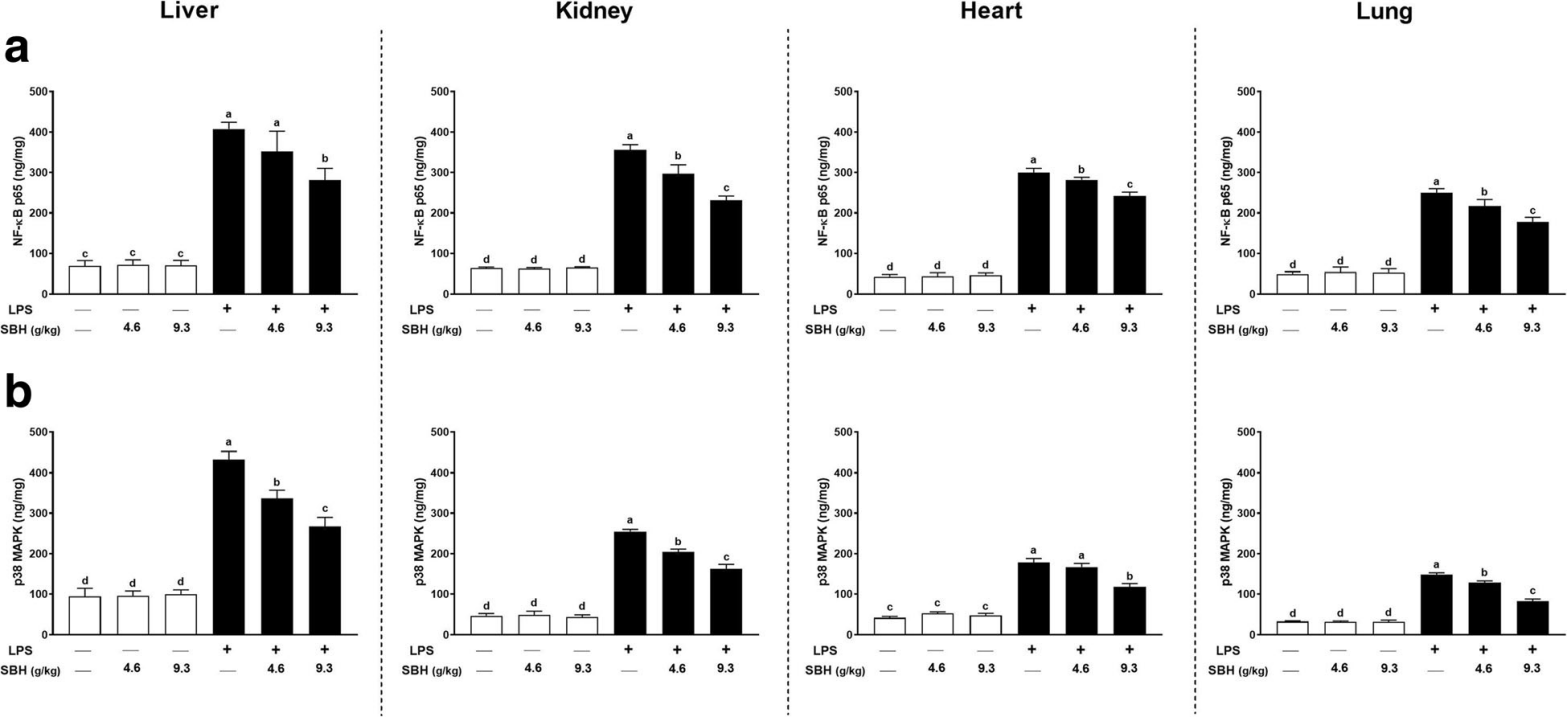
SBH suppresses (**a**) NF-κB p65 and (**b**) p38 MAPK in liver, kidney, heart and lung of LPS-induced rats. Data are mean ± standard error of the mean (*n* = 6). Significantly different values are indicated by different superscripts

NF-κB p65 (Fig. 6A) and p38 MAPK (Fig. 6B) were assayed in the tissue homogenate of liver, kidney, heart and lung of rats. The results showed significant (*P* < 0.05) increase in both NF-κB p65 (Fig. 6A) and p38 MAPK (Fig. 6B) levels in all targeted organs of LPS-induced rats when compared with the control group. Treatment of the LPS-induced rats with SBH remarkably decreased the levels of NF-κB p65 and p38 MAPK levels. However, the effect of 4.6 g/kg SBH on liver NF-κB p65 and heart p38 MAPK levels was non-significant when compared with the LPS control group.

Both doses of SBH didn’t exert a significant effect on the levels of NF-κB p65 and p38 MAPK in liver, kidney, heart and lung of normal rats (Fig. 6).

### Effect of SBH on oxidative stress markers and antioxidants in LPS-induced rats

**Fig. 7:**
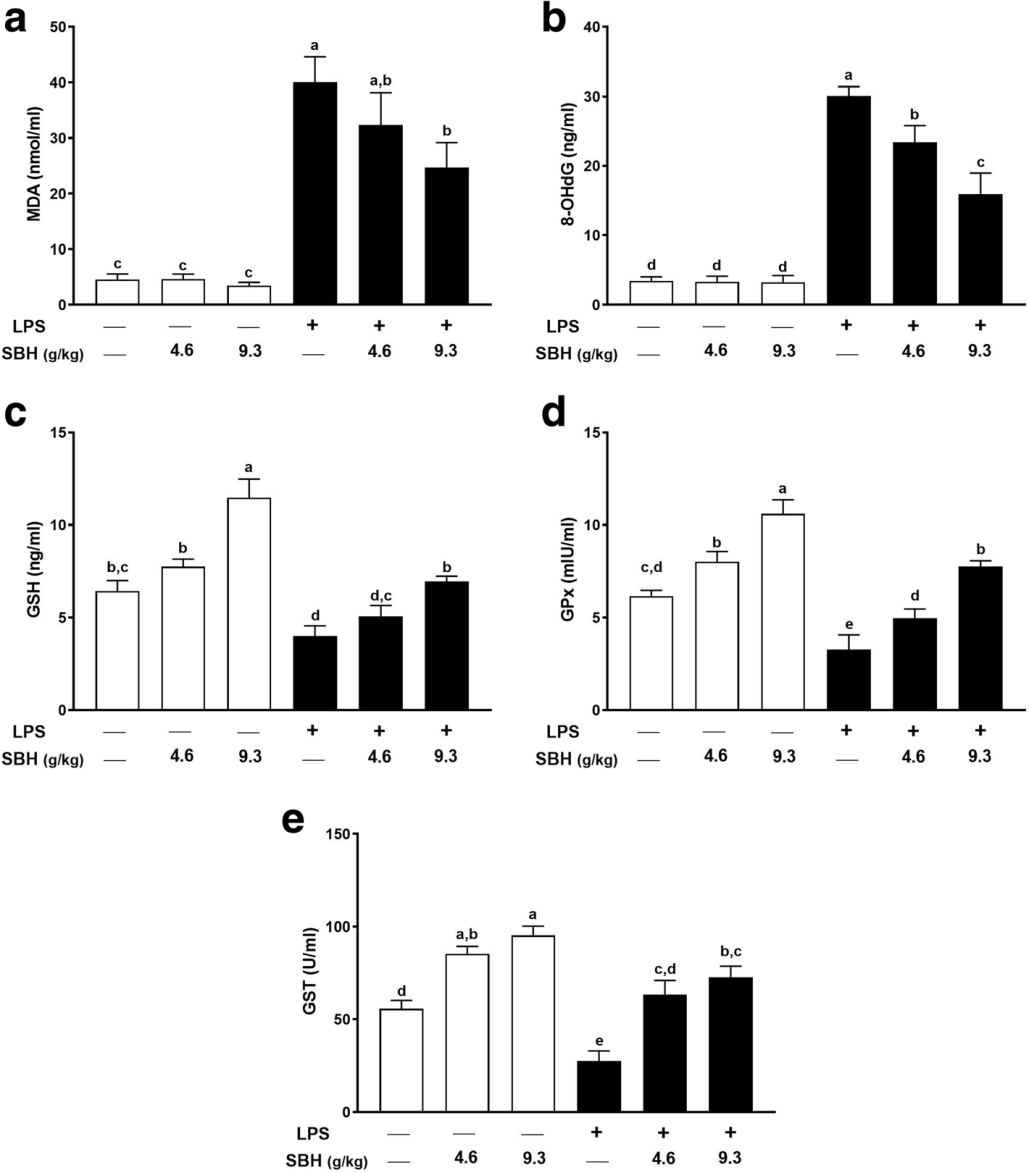
SBH attenuates LPS-induced (**a**) lipid peroxidation and (**b**) oxidative DNA damage, and enhances the antioxidants (**c**) GSH, (**d**) GPx and (**e**) GST in control and LPS-induced rats. Data are mean ± standard error of the mean (*n* = 6). Significantly different values are indicated by different superscripts

The involvement of antioxidant efficacy of SBH in attenuating CSSI was determined through quantifying the antioxidant enzymes and oxidative stress markers in LPS-induced rats. As presented in Fig. 7, a remarkable increase in MDA and 8-OHdG along with a significant decrease in GSH, GST and GPx were observed in LPS group when compared with the control group. On the other hand, daily supplementation of SBH significantly ameliorated MDA (Fig. 7A), 8-OHdG (Fig. 7B), GSH (Fig. 7C), GPx (Fig. 7D) and GST (Fig. 7E) in LPS-induced rats, whereas exerted no effect in normal rats.

### Effect of SBH on Nrf2 in liver, kidney, heart and lung of control and LPS-induced rats

**Fig. 8:**
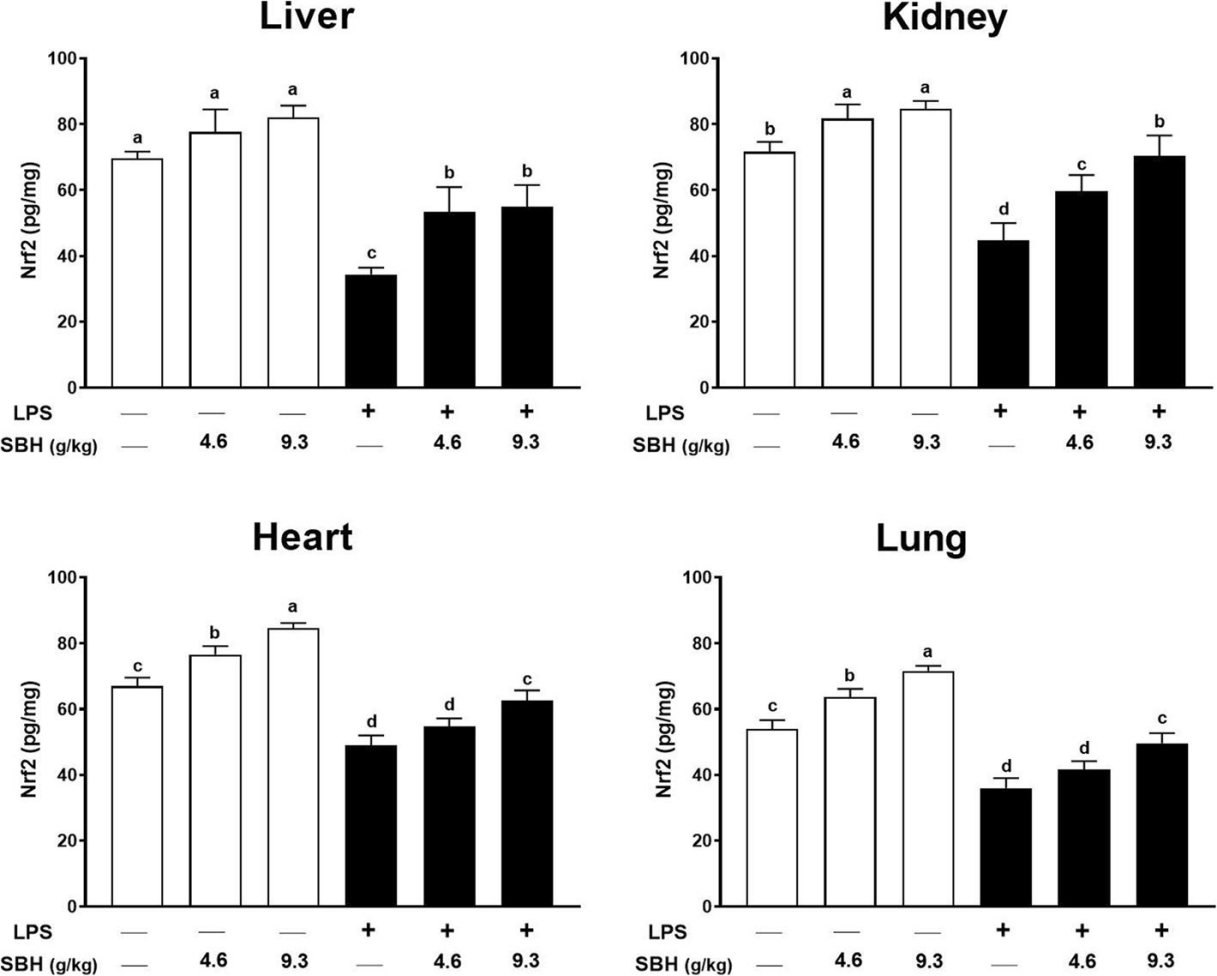
SBH up-regulates Nrf2 in the liver, kidney, heart and lung of normal and LPS-induced rats. Data are mean ± standard error of the mean (*n* = 6). Significantly different values are indicated by different superscripts

Nrf2 is a redox-sensitive transcription factor that control the expression of several antioxidant and defense proteins [38–41]. Therefore, we determined the expression levels of Nrf2 in liver, kidney, heart and lung of normal and LPS-induced rats treated with SBH (Fig. 8). LPS administration significantly decreased the levels of Nrf2 in liver, kidney, heart and lung of rats when compared with the control group. In contrast, SBH administration significantly alleviated the levels of Nrf2 in both the liver and kidney of LPS-induced rats. Although the 4.6 g/kg SBH didn’t increase Nrf2 levels in the heart and lung of LPS-induced rats, the 9.3 g/kg dose produced a significant amelioration. Surprisingly, treatment of the normal rats with either dose of SBH significantly increased Nrf2 levels in kidney, heart and lung and showed a trend increase in the liver.

### Effect of SBH on liver, kidney and heart function markers in LPS-induced rats

**Fig. 9:**
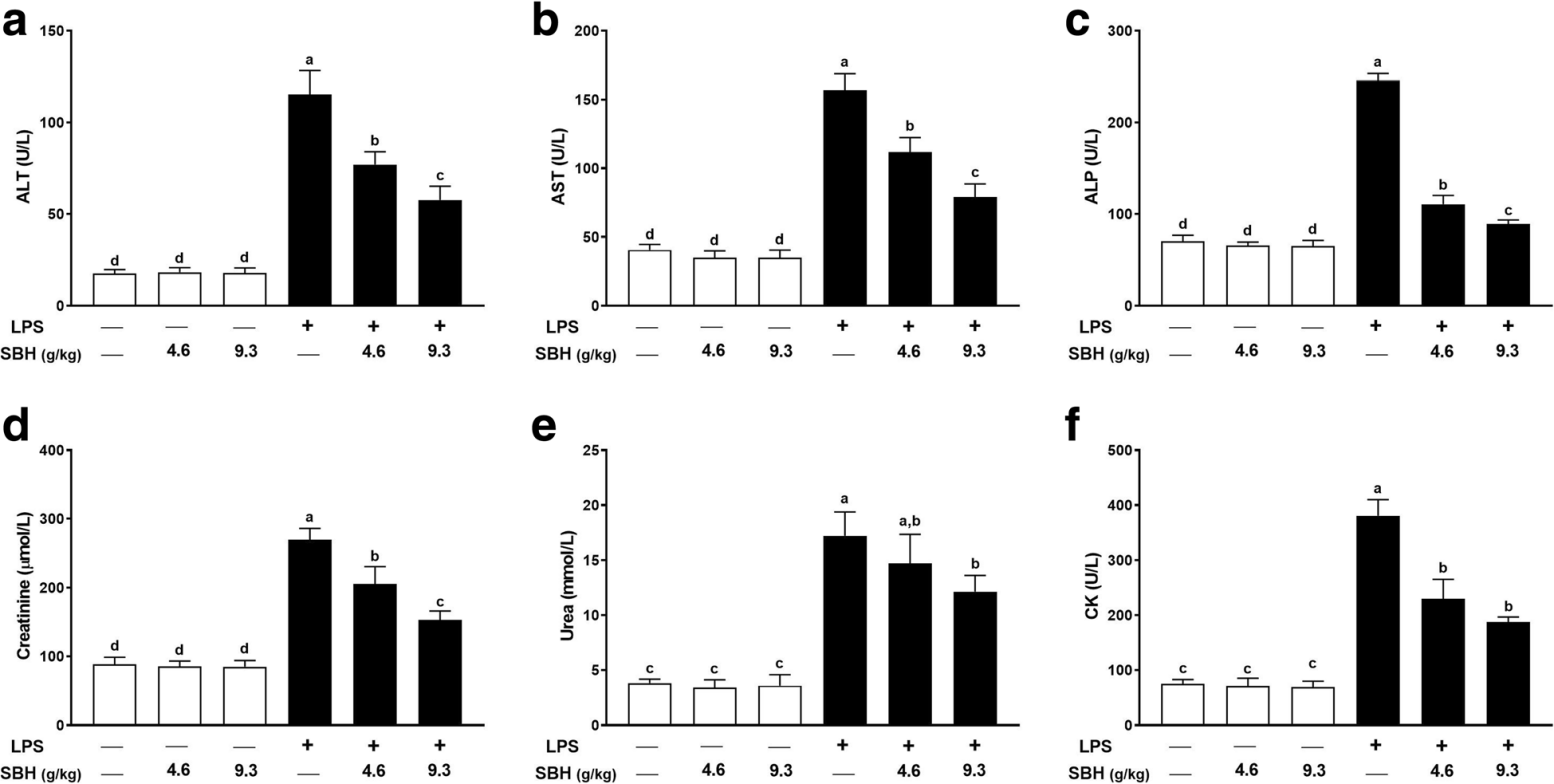
SBH alleviates serum levels of (**a**) ALT, (**b**) AST, (**c**) ALP, (**d**) creatinine, (**e**) urea and (**f**) CK in LPS-induced rats. Data are mean ± standard error of the mean (*n* = 6). Significantly different values are indicated by different superscripts

The assessment of liver, kidney and heart function and injury was determined by measuring serum levels of ALT, AST, ALP, creatinine, urea and CK. As represented in Fig. 9, LPS-induced rats exhibited a significant (*P < 0.05*) increase in serum ALT (Fig. 9A), AST (Fig. 9B), ALP (Fig. 9C), creatinine (Fig. 9D), urea (Fig. 9E) and CK (Fig. 9E) when compared with the control rats. Treatment of the LPS-induced rats with SBH significantly ameliorated serum levels of the liver, kidney and heart function markers. In contrast, oral supplementation of SBH didn’t alter these markers in serum of normal rats.

### Effect of SBH on LPS-induced histopathological changes in rats

**Fig. 10:**
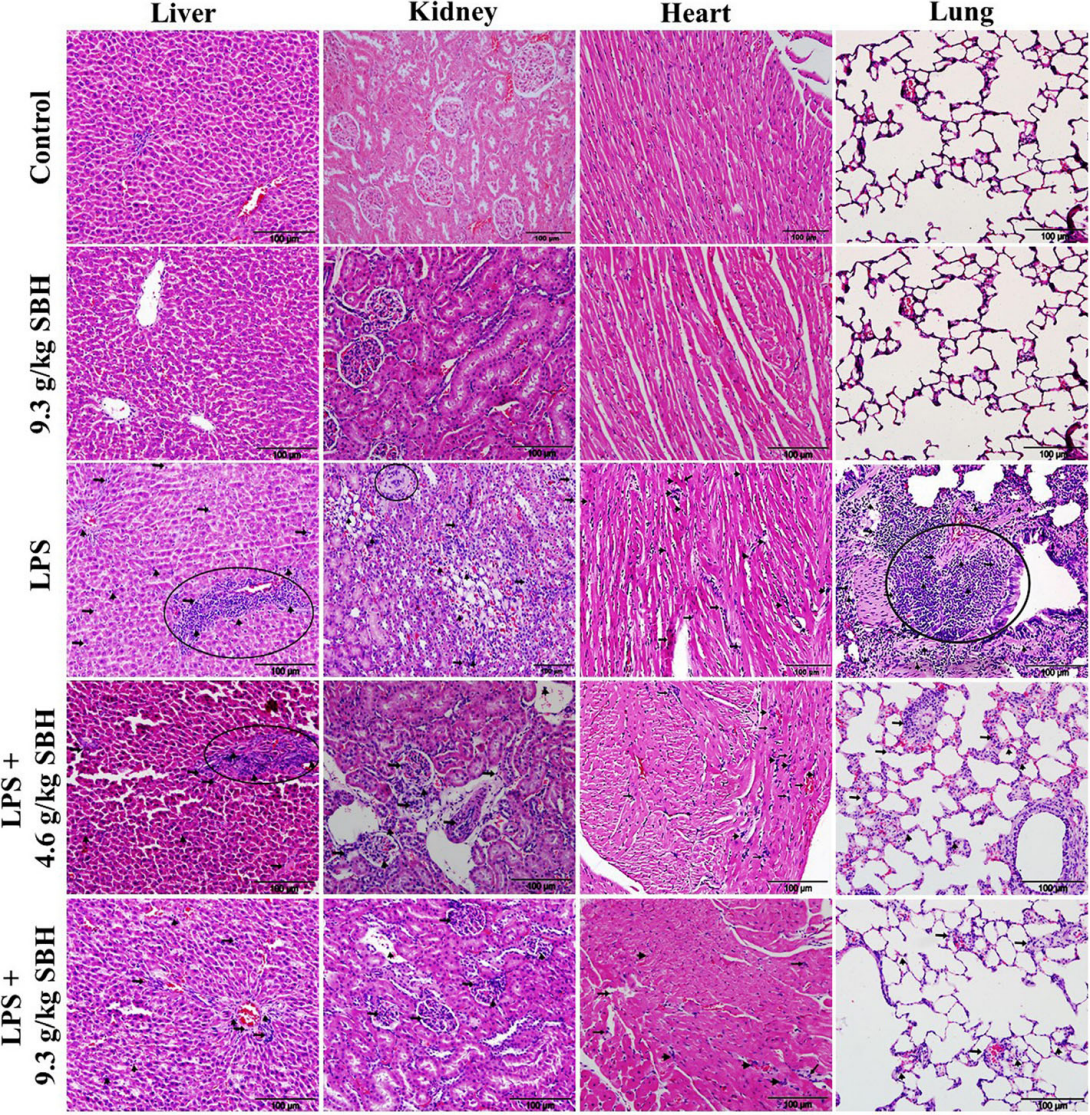
SBH attenuates histological alterations in the liver, kidney, heart and lung of LPS-induced rats. Microscopic examination of H&E-stained sections of the control and 9.3 g/kg SBH-supplemented rats revealed normal histological structure of the liver, kidney, heart and lung. In contrast, liver sections of the LPS-induced rats showed an increase in lymphocytes (arrow heads) and neutrophils (arrows) infiltration and necrosis (circle). LPS-induced rats received 4.6 or 9.3 g/kg SBH showed a significant reduction in lymphocyte (arrow heads) and neutrophils (arrows) infiltration and improved liver histology. The kidney of LPS-induced rats showed infiltration of lymphocytes (arrow heads) and neutrophils (arrows), renal granuloma (circle) and degeneration of tubular cells. SBH-supplemented LPS-induced rats showed significantly reduced infiltration of lymphocytes (arrow heads) and neutrophils (arrows). The heart sections of LPS-induced rats showed degeneration of cardiomyocytes and infiltration of lymphocytes (arrow heads) and neutrophils (arrows), and the lung sections revealed edema, alveolar wall thickness and lymphocytes (arrow heads) and neutrophils (arrows) infiltration. Treatment of the LPS-induced rats with 4.6 or 9.3 g/kg SBH resulted in reduced infiltration of lymphocytes (arrow heads) and neutrophils (arrows) and improved histological appearance of the heart and lung tissues

**Fig. 11:**
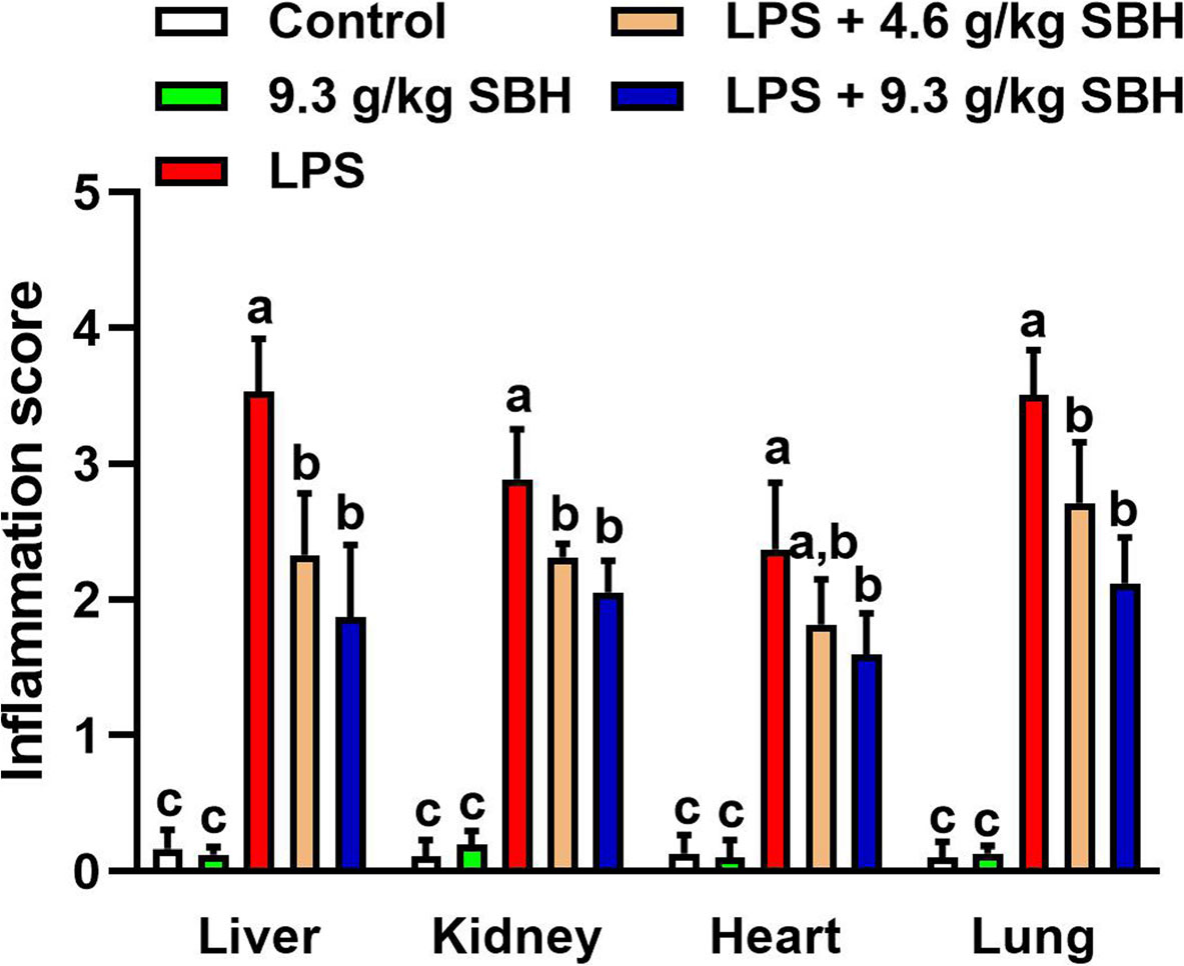
Inflammation score of the liver, kidney, heart and lung of SBH-supplemented control and LPS-induced rats. Data are mean ± standard error of the mean (*n* = 6). Significantly different values are indicated by different superscripts

Microscopic examination of the H&E-stained sections in the liver of control and SBH-supplemented rats showed normal structure of the hepatic lobules, hepatocytes and sinusoids (Fig. 10). In contrast, LPS-induced rats exhibited several histopathological changes, including infiltration of lymphocytes and neutrophils, necrosis and other manifestations (Fig. 10), as well as, significantly increased inflammation score when compared with the control rats (Fig. 11). Oral supplementation of SBH resulted in attenuation of the inflammatory cell infiltration (Fig. 10) and significantly reduced inflammation score when compared with the LPS control group (Fig. 11).

H&E-stained sections in the kidney of LPS-induced rats showed significant degenerative changes, including degeneration of tubular cells, modest glomerular atrophy, granuloma, blood cells extravasating into the tubulointerstitial spaces and aggregation of inflammatory cells (Fig. 10). In addition, LPS-induced rats exhibited significantly increased inflammation score in the kidney when compared with the control group (Fig. 11). The kidney of LPS-induced rats treated with SBH showed a significant attenuation in inflammatory cells infiltration (Fig. 10) and inflammation score (Fig. 11).

Microscopic examination of the H&E-stained sections in the heart revealed significant changes during the experimental period, as presented in Fig. 10. The inflammatory and degenerative changes in LPS-induced rats are manifested in neutrophils and lymphocytes infiltration along with cardiomyocyte damage. These pathological features were attenuated in SBH-treated LPS-induced rats as showed by the decreased infiltration of neutrophils and lymphocytes, and the significantly reduced inflammation score when compared with the LPS control group (Fig. 11).

The histological examination of lung tissues of the LPS-induced rats demonstrated significant infiltration of inflammatory leukocytes into alveolar and interstitial spaces, along with edema and tissue destruction (Fig. 10). When compared with the control group, LPS-induced rats showed a significant increase in inflammation score (Fig. 11). SBH treatment reduced the inflammatory cells infiltration and tissue damage in the lung of LPS-induced rats (Figs. 10 & 11).

## Discussion

CSSI, also known as low-grade chronic inflammation, has been considered as the major link in the pathogenesis of several noncommunicable diseases, including type 2 diabetes, obesity, metabolic syndrome, osteopenia, and neurodegenerative and cardiovascular diseases [8, 10, 11, 13]. Herein, a model of LPS-induced male rats was appointed successfully to investigate, for the first time, the impact of SBH supplementation on CSSI. Oral supplementation of SBH remarkably improved body weight, food intake and the survival rate of LPS-induced rats. In addition, SBH ameliorated LPS-induced leukocytosis, neutrophilia, lymphocytosis and monocytosis, indicating its potent anti-inflammatory efficacy. In support of these findings, SBH decreased the circulating levels of CRP, TNF-α, IL-1β, IL-6, IL-8 and MCP-1, and diminished NF-κB and p38 MAPK signaling in different tissues of LPS-induced rats. Simultaneously, SBH enhanced antioxidant defenses, up-regulated Nrf2, and attenuated lipid peroxidation and oxidative DNA damage in LPS-induced rats. Therefore, SBH prevented LPS-induced CSSI via its dual ability to attenuate inflammation and oxidative stress.

LPS has been widely used both in vitro and in vivo to evaluate the anti-inflammatory potential of many agents [42], and long-term administration of LPS can mimic chronic inflammation diseases [43]. In addition, exploring the low-dose influx of LPS to illustrate the etiology of chronic inflammatory conditions has been justified by the plentiful presence of LPS in the surrounding [8]. Therefore, many experimental studies have used LPS to induce chronic subclinical inflammation either by intermittent injection or by using osmotic pump release [44, 45]. In line with these reports, the current study demonstrated that the selected dose and administration frequency of LPS have successfully produced the typical features of CSSI, including partial suppression of food intake and body weight gain, leukocytosis and elevated pro-inflammatory mediators in the peripheral circulation. The daily consumption of SBH ameliorated the impact of CSSI on animal performance through improving food intake and maintaining a normal body weight gain. Consuming functional foods rich in bioactive compounds such as polyphenols has been well-documented to preserve hemostasis during low-grade chronic inflammation [46]. In addition, the long-term consumption of SBH has been proven previously to maintain normal food intake and body weight gain [47].

CSSI is associated with activation of NF-κB and p38 MAPK which represent the key signaling pathways involved in regulating inflammation. NF-κB is a redox-sensitive transcription factor that is activated by ROS, leading to increased production of inflammatory cytokines, including IL-1β, IL-6, TNF-α and others. In addition, ROS have been implicated in the activation of p38 MAPK [48], a kinase that regulates the production of IL-1β and TNF-α [49]. Hence, attenuation of NF-κB and p38 MAPK signaling can suppress the production of pro-inflammatory cytokines. Interestingly, our results demonstrated that oral supplementation of SBH down-regulated both NF-κB and p38 MAPK in the liver, kidney, heart and lung, and ameliorated leukocytosis and serum inflammatory mediators in LPS-induced rats. These results are consistent with recent studies showing the anti-inflammatory activities of honey and its constituents [50–54]. Accordingly, propolis, a resinous mixture of bee saliva and beeswax, has down-regulated NF-κB and p38 MAPK in LPS-stimulated Raw 246.7 cells. The effect of propolis was attributed to the presence of hydroxycinnamic acids [51]. It is worth mentioning that hydroxycinnamic acids, including caffeic and coumaric acids have been identified in SBH [22] and have shown to alleviate inflammation through modulating NF-κB and p38 MAPK pathways [51]. Caffeic acid phenethyl ester, the principal component of propolis, has been recently reported to suppress inflammation in the brain of hexavalent chromium-induced rats [55]. In addition, supplementation of Gelam honey improved the survival of animals and improved antioxidants after 24 h of LPS administration [50].

Leukocytosis has been considered as an indicator of CSSI [56], and other chronic diseases, such as, type 2 diabetes, cardiovascular and neurodegenerative diseases and metabolic syndrome [57–60]. Here, frequent LPS administration increased WBCs number which could be attributed to increased production of leukocytes from bone marrow [61]. The plentiful presence of macrophages and lymphocytes during CSSI is due to the ongoing stimulation and the increased production of chemokines and chemoattractant factors by immune cells and capillary endothelium which subsequently facilitate immune cell infiltration into the tissues [62]. In this context, microscopic examination of the liver, kidney, heart and lung tissues of LPS-induced rats revealed a significant infiltration of inflammatory leukocytes. Additionally, increased neutrophil-lymphocyte ratio (NLR) has been considered as a prognostic tool for CSSI and a predictor of survival in different chronic conditions [63]. In contrast, it has been documented that diet rich in polyphenols and/or fish oil normalized the absolute WBCs count and NLR through reducing the release of chemoattractant factors [64]. In our study, LC-ESI-MS/MS analysis showed the rich polyphenol content of SBH and this can explain, at least in part, the ameliorative potential of SBH on LPS-induced leukocytosis, inflammation and tissue damage. In addition to amelioration of leukocytosis, SBH reduced the circulating levels of inflammatory mediators, including CRP, MCP-1, TNF-α, IL-1β and IL-6 in LPS-induced rats.

Elevated serum levels of CRP are associated with different chronic inflammatory diseases, including coronary heart disease, rheumatoid arthritis and cancer [65]. CRP is released by hepatocytes in response to pro-inflammatory cytokines and functions to activate the classical complement pathway which in turn prevent autoimmunity and defend against infection [66]. CSSI has been reported to be associated with elevated levels of CRP [15]. SBH supplementation remarkably ameliorated serum CRP levels in LPS-induced rats which could be a direct result of its high content of phenolic compounds and flavonoids [22]. The dietary intake of flavonoids has been inversely associated with CRP levels among adults [67], and supplementation of honey for 12 weeks was reported to normalize CRP in chronic smokers [53]. Similarly, the dietary intake of honey for 15 days by diabetic and hyperlipidemic subjects was associated with lower levels of CRP [68]. Furthermore, MCP-1 plays a key role in developing CSSI via increasing the recruitment of bone-derived monocytes, stimulating macrophage cell division and enhancing macrophage infiltration into tissues [69]. Therefore, amelioration of MCP-1 levels by SBH might have a significant role in the suppressed infiltration of leukocytes into the liver, kidney, heart and lung of LPS-induced rats in the present investigation.

Given the role of ROS in the activation of NF-κB and p38 MAPK and the subsequent release of pro-inflammatory cytokines, oxidative stress is implicated in the pathophysiological changes during CSSI. Accordingly, LPS-induced rats showed a significant increase in lipid peroxidation and oxidative DNA damage, accompanied with diminished antioxidant defenses. In addition, the redox-sensitive Nrf2 was significantly down-regulated in the liver, kidney, heart and lung of LPS-induced rats, demonstrating excessive production of ROS and oxidative stress. Nrf2 is a transcription factor that regulates the gene expression of endogenous phase II detoxifying and antioxidant enzymes. Nrf2 has been thought to be a therapeutic target for ameliorating chronic low-grade inflammation [70, 71]. Recent studies have shown that Nrf2 activation is associated with attenuation of oxidative stress and enhancement of antioxidant defenses in liver, kidney, brain and endothelial cells [72–79]. Interestingly, SBH treatment increased the protein levels of Nrf2 in liver, kidney, heart and lung of the LPS-induced rats. Consequently, LPS-induced rats treated with SBH exhibited reduced lipid peroxidation and oxidative DNA damage, and enhanced antioxidant defenses. More interestingly, SBH up-regulated Nrf2 in normal rats. Hence, it is suggested that the regular supplementation of SBH promotes the endogenous antioxidant system. In addition, the anti-inflammatory effect of SBH could be connected to its ability to up-regulate Nrf2. In this context, the lack of Nrf2 in mouse primary astrocytes augmented the production of inflammatory cytokines [80]. Furthermore, these pharmacological actions were further confirmed in the histological sections of liver, kidney, heart and lung where SBH attenuated the infiltration of inflammatory leukocytes and prevented tissue damage.

Limitations in nutritional biochemistry research are unavoidable matters due to several challenges, such as, limited time, confounding factors and the high cost of arrays. One of the limitations in the current study is the seasonal collection and floral type of SBH which in turn can affect its phenolic profile and subsequently the pharmacological actions. Another limitation in this research is that anti-inflammatory mediators and adhesion molecules were not measured.

## Conclusions

**Fig. 12:**
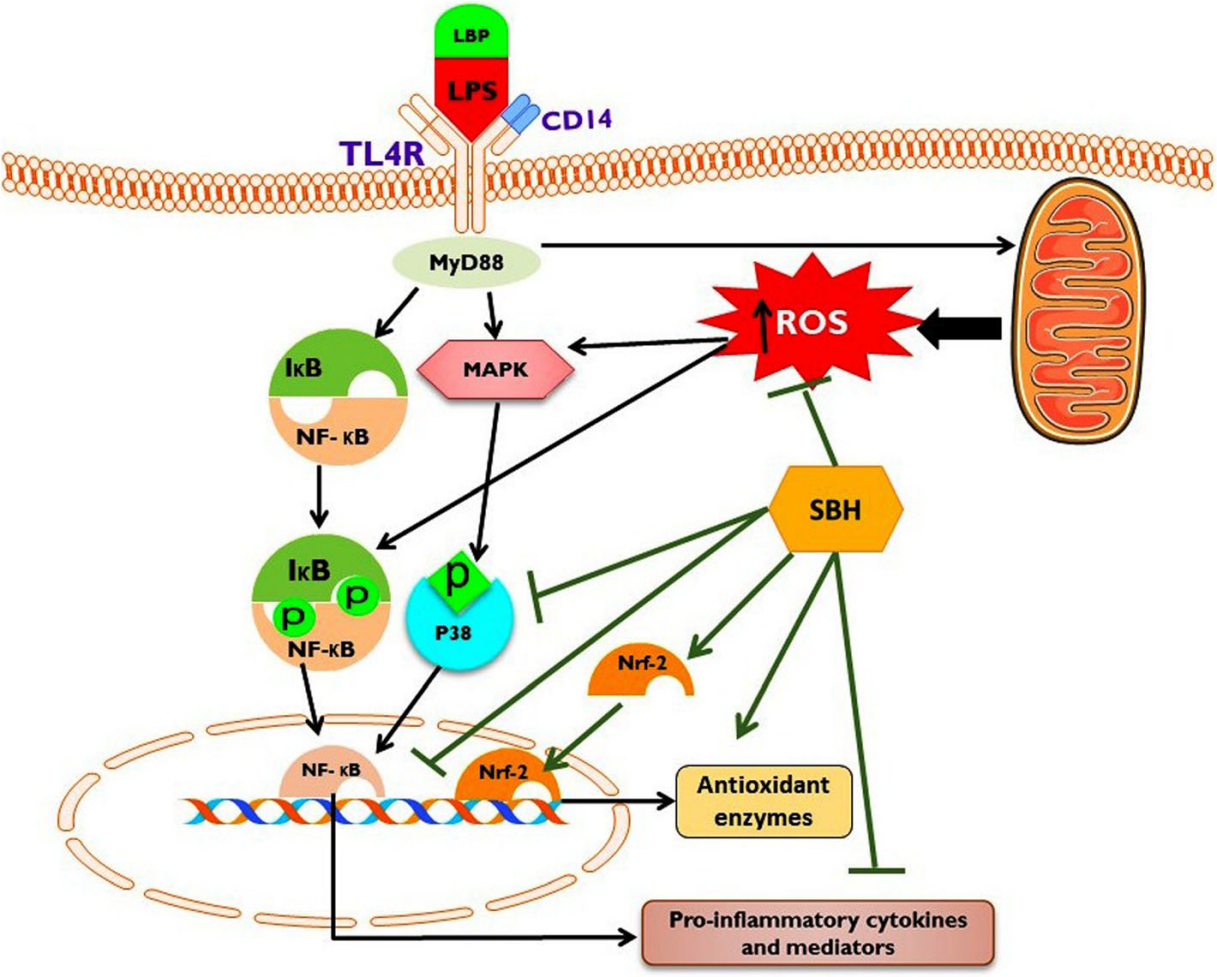
The proposed mechanism underlying the ameliorative effect of SBH on LPS-induced CSSI induced. The binding of LPS with Toll-like receptor 4 (TLR-4) provokes the production of reactive oxygen species (ROS) in the mitochondria, and activates p38 mitogen activated protein kinase (p38 MAPK) and nuclear factor-kappaB (NF-κB), resulting in the production of inflammatory cytokines and tissue damage. SBH up-regulates nuclear factor (erythroid-derived 2)-like 2 (Nrf2), enhances antioxidant defenses, attenuates ROS production, and suppresses NF-κB and p38 MAPK signaling, leading to diminished inflammation. Therefore, SBH prevented LPS-induced CSSI via its dual ability to attenuate inflammation and oxidative stress

The results reported in this investigation demonstrated that SBH supplementation conferred protection against CSSI through attenuating oxidative stress and inflammation. The anti-inflammatory and antioxidant efficacies of SBH are mediated via up-regulation of Nrf2 and suppression of NF-κB and p38 MAPK signaling (a mechanistic pathway is represented in Fig. 12). These findings indicate that SBH could be a potential candidate for ameliorating and preventing CSSI and related chronic diseases.
